# Validation of data quality in the Swedish National Register for Breast Cancer

**DOI:** 10.1186/s12889-019-6846-6

**Published:** 2019-05-02

**Authors:** Lars Löfgren, Sandra Eloranta, Kamilla Krawiec, Annette Asterkvist, Charlotta Lönnqvist, Kerstin Sandelin, On behalf of the steering group of the National Register for Breast Cancer

**Affiliations:** 1 Department of Surgery, S:t Görans Hospital, SE-11281 Stockholm, Sweden; 2 Scandinavian Development Services, Danderyd, Sweden; 3 Regional Cancer Centre Stockholm – Gotland, Stockholm, Sweden; 4 Department of Molecular Medicine and Surgery Karolinska Institutet, Stockholm, Sweden

**Keywords:** Breast cancer, Quality register, Validation

## Abstract

**Background:**

The National Breast Cancer Register (NBCR) of Sweden was launched in 2008 and is used for quality assurance, benchmarking, and research. Its three reporting forms encompass Notification, Adjuvant therapy and Follow-up. Target levels are set by national and international guidelines. This national validation assessed data quality of the register.

**Methods:**

Data recorded through the Notification form were evaluated for completeness, timeliness, comparability and validity. Completeness was assessed by cross-linkage to the Swedish Cancer Register (SCR). Comparability was analyzed by comparing registration routines in NBCR with national and international guidelines. Timeliness was defined as the difference between the earliest date of diagnosis and the reporting date to NBCR. Validity was assessed by re-abstraction of medical chart data for 800 randomly selected patients diagnosed in 2013.

**Results:**

The completeness of the NBCR was high with a coverage across regions and years (2010–2014) of 99.9%. Of all incident cases reported to the NBCR in 2013 (*N* = 8654), 98.5% were included within 12 months and differences between health regions were essentially negligible. Coding procedures followed guidelines and were uniformly adhered to. The proportion of missing values was < 5% for most variables and reported information generally had high exact agreement (> 90%).

**Conclusions:**

Completeness of data, comparability and agreement in the NBCR was high. For clinical quality purposes and benchmarking, improved timeliness is warranted. Assessment of validity has resulted in a thorough review of all variables included in the Notification form with clarifications and revision of selected variables.

## Background

The national cancer inquiry in 2005 concluded that cancer care in Sweden, although keeping a high standard, had several inequities both in its structure, its process and in outcome [1]. Regional cancer centers were established through the Association of Local Authorities and Regions, for buildup of national cancer registers [2]. For the most prevalent cancers, regional registers were already established and formed the basis for many outcome studies.

The National Breast Cancer Register (NBCR) has been operating since 2008 and collects data in a national common database. It encompasses the diagnostic and therapeutic processes and outcome for all primary invasive and in situ breast cancer cases. Registration is performed via the web-based INCA platform (Information Network for Cancer care). Quality indicators proposed by the National Board of Health and Welfare mirror the care process. Coding routines follow national and international classification guidelines. Cancer staging and TNM classification followed the AJCC Cancer Staging Manual 7: th edition and the TNM Classification of Malignant Tumours, UICC 7: th edition [3, 4].

The register consists of three sections, Notification (including planned adjuvant therapy), Adjuvant therapy and Follow-up. Target levels are set by national and international guidelines. Continuous revisions and updates of the variables makes it a dynamic work tool. The responsibility for reporting lies on the individual health care providers and data are further monitored by the six Regional Cancer Centers located across Sweden’s health care regions. The NBCR steering committee has national multi-professional and multi-disciplinary team members and representatives from the breast cancer survivor group. Individuals can actively opt out from registration although this is extremely rare.

Based on register data, the National Board of Health and Welfare has previously published reports for several cancer diagnoses that assess and follow-up on defined quality indicators such as compliance and timeliness [5]. The reports serve as audits, quality assurance and benchmarks. Other stakeholders such as the public, patient representatives, purchasers of health care and decision-makers make use of reported data. Register data also provides a resource for clinical and epidemiologic research.

In 2013, the NBCR steering committee decided to conduct a nationwide validation of the recorded data based on a manual (AKI) developed by the working group for quality registers and INCA [6]. The manual builds upon the validation strategy of cancer registry data proposed by Parkin and Bray [7, 8] and includes the following four quality dimensions; timeliness, completeness, comparability and validity. This study presents the results of the nationwide validation of the NBCR and aims to describe how the results have been instrumental for improving the register through revision of the included variables, the reporting forms and its manual, and to assist in training of data managers.

## Methods

For evaluation of timeliness, all incident cases reported to the NBCR in 2013 were included (*N* = 8654), and the difference in time between the earliest date of diagnosis and the reporting date in the registry was calculated.

Completeness was assessed by comparing the cases in the NBCR with registrations in the Swedish Cancer Registry (SCR) [9], to which reporting is mandatory according to the National Board of Health and Welfare’s regulations (SOSFS2006:15). Data from the time period 2010–2014 was used. The completeness of the SCR is secured as any diagnosed cancer case is reported by the clinician and from the pathology laboratory after verification of morphological examinations i e biopsies and autopsy. Two publications describe in detail the process [10, 11].

Comparability refers to the recording and coding practices and should be clear, nationally uniform and follow international guidelines to enable comparisons between regions and countries. Inclusion criteria are: location (primary breast cancer); sex (women and men); age (all ages); morphology (invasive breast cancer and carcinoma in situ); basis for diagnosis (all cases except diagnosis at autopsy).

Two control functions secure comparability. Firstly, the manual and the report form are unique documents. Secondly, monitoring is performed at the regional cancer centers whereby adherence to inclusion criteria and or any erroneously reported data and or ambiguity will be corrected.

Comparability concerning the workflow was assessed by a questionnaire addressing how different breast units handled reporting routines, involved staff, time allotted, and management support [12].

To assess validity, re-abstracted data from medical records was compared to the reported data via an independent review process. Eight hundred recorded cases between September 2013 and January 2014, were randomly selected using a two-stage cluster sampling plan.

Two hospitals offering breast cancer services (ranked according to size) from each health care region were selected. Within each region (cluster), a subsample of all breast cancer patient records in the 12 selected hospitals were drawn with a probability proportional to the size of region and hospital. The sampling plan was chosen to ensure national representation as well as participation from both large and small breast cancer units.

Re-abstraction of medical records took place in the second part of 2014 and was performed by three specialist nurses with previous experience in register validation and monitoring, henceforth referred to as validators. The re-abstracted information was entered into a specially designed module and subsequently merged with the originally recorded data to calculate exact data agreement. Exact agreement corresponds to the proportion of women for whom the data recorded in the NBCR is the same as in the validation data set. Missing observations were also included in the calculations of exact agreement to account for the plausible situations when 1) data had been reported to the NBCR but could not be found in the medical records, 2) the information was available in the medical records but had not been reported to the NBCR. Strength of agreement was measured by Cohen’s Kappa (κ) scores for categorical variables, including 95% confidence intervals (CI), and Pearson correlation coefficients (r) for numerical variables.

## Results

### Timeliness

**Fig. 1:**
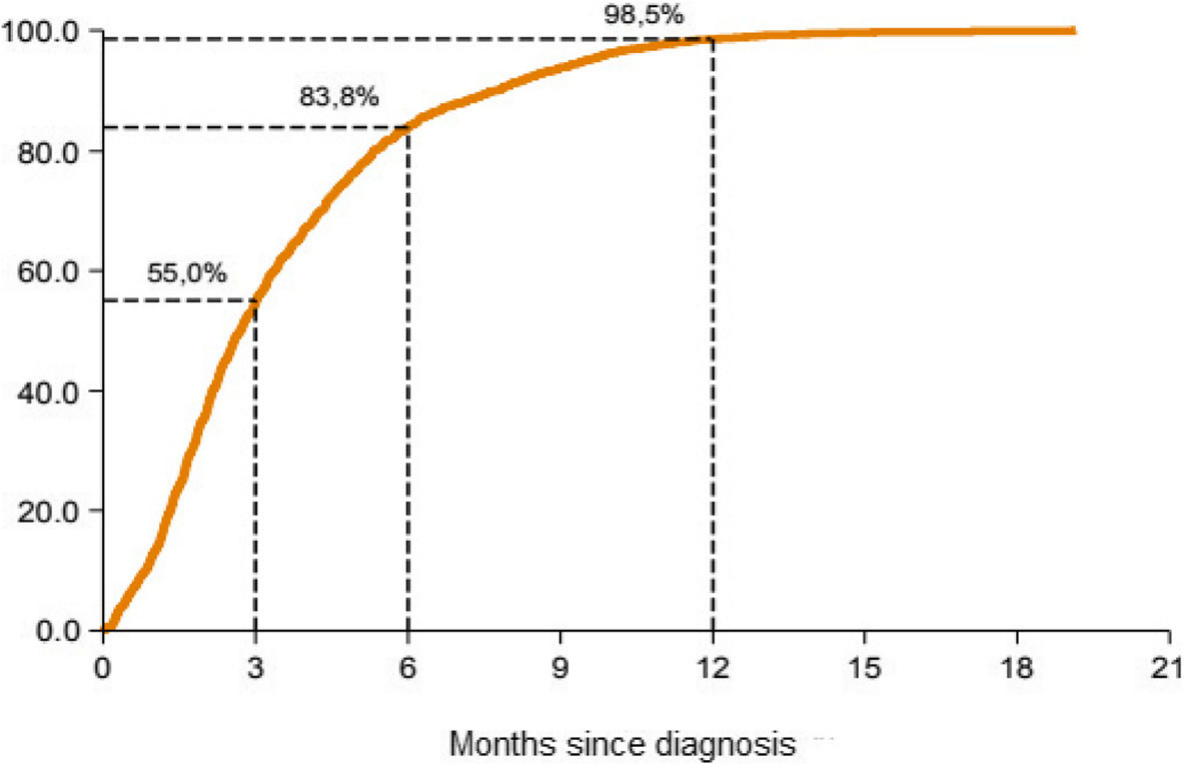
Timeliness of breast cancer registration in Sweden 2013. Displayed as the proportion of all new breast cancer diagnoses reported to the National Breast Cancer Register (NBCR) by elapsed time since the diagnosis (in months)

Timeliness of reporting showed wide regional variations within 3 months, ranging from 30.2% in the Southeast to 77.4% in the Uppsala-Örebro regions. In 2013, 83.8% of all incident cases had been reported to NBCR within 6 months. At 12 months, 98.5% had been reported (Fig. 1). Differences between the regions were essentially negligible after 1 year (Table 1).

**Table 1:** Timeliness of reporting to the National Breast Cancer Register (NBCR) by health care region in Sweden in 2013. Percentage of cases reported after three respectively 6 and 12 months

Health care region	% after 3 months	% after 6 months	% after 12 months
North	49.1	89.2	99.3
Stockholm-Gotland	40.5	70.7	99.2
Southeast	30.2	87.0	99.4
Uppsala-Örebro	77.4	90.9	98.8
South	57.9	82.9	96.7
West	58.4	86.6	98.2

### Completeness

The average coverage across all healthcare regions during 2010–2014 was 99.9%. The difference in coverage between regions was small for all calendar years (Table 2).

**Table 2:** Completeness (%) of reporting to the National Breast Cancer Register (NBCR) 2010–2014 as compared to reporting to the National Cancer Register by health care region in Sweden

Year of diagnosis	Sweden	North	Stockholm-Gotland	Southeast	Uppsala-Örebro	South	West
2010	99.9	100	99.9	99.9	99.9	100	99.9
2011	99.9	100	99.9	99.9	99.9	98.9	99.8
2012	99.8	99.8	99.9	99.9	99.8	98.1	99.7
2013	99.9	99.9	99.9	99.8	100	98.6	99.8
2014	99.9	99.3	99.5	99.7	99.4	93.4	96.5

### Comparability

Eleven of 12 units responded to the questionnaire about the workflow in the reporting process. Nurses and doctors were mainly responsible for reporting to the NBCR, and responses indicated local and regional differences concerning the workflow and routines. Notable is that concerning allotted time resources for registration, five responded, affirmative and six negating. Weather support from the departmental leadership prevailed the responders described a strong formal support but a weak actual support and insufficient resource allocation.

### Validity

Of the 800 patients selected for re-abstraction of medical records, one individual had been incorrectly registered and was excluded. The number of missing observations, the exact agreement between the originally recorded and the re-abstracted data is summarized in Table 3.

**Table 3:** Missing data and exact agreement for variables included in the validation of the notification section of the Swedish National Register for Breast Cancer

Variable in the Swedish National Register for Breast Cancer		Missing values in the NBCR (%)	Missing values in the medical records (%)	Missing values in both sources (%)	Exact agreement (%)
Diagnostics	Menstrual status	62/799 (7.8)	90/799 (11.3)	28/799 (3.5)	82.6
	Laterality	0/799 (0)	0/799 (0)	0/799 (0)	98.5
	Initial contact (referral/mammography/self-admission)	14/799 (1.8)	7/799 (0.9%)	0/799 (0)	88.1
	Malignant diagnosis confirmed at first visit	3/799 (0.4)	29/799 (3.6)	0/799 (0)	83.5
	Screening detected	5 /799 (0.6)	3/799 (0.4)	0/799 (0)	94.9
	Malignant diagnosis confirmed preoperatively	4/799 (0.5)	43/799 (5.4)	2/799 (0.3)	91.2
	Base for diagnosis	0/799 (0)	0/799 (0)	0/799 (0)	77.9
	Preoperative multidisciplinary conference	2/799 (0.3)	2/799 (0.3)	0/799 (0)	95.2
Stage	T classification	1/799 (0.01)	0/799 (0)	0/799 (0)	70.1
	N classification	0/799 (0)	0/799 (0)	0/799 (0)	93.0
	M classification	0/799 (0)	0/799 (0)	0/799 (0)	98.6
Planned care	Primary surgery planned	0/799 (0)	0/799 (0)	0/799 (0)	98.3
	Reason for no primary surgery	1/96 (1.0)	5/96 (5.2)	1/96 (1.0)	53.1
	Primary systemic treatment	5/799 (0.6)	2/799 (0.3)	0/799 (0)	95.6
	Primary radiotherapy	0/69 (0)	0/69 (0)	0/69 (0)	100
	Primary endocrine therapy	0/69 (0)	0/69 (0)	0/69 (0)	95.7
	Primary chemotherapy	0/69 (0)	0/69 (0)	0/69 (0)	97.1
	Other primary systemic or targeted treatment	0/69 (0)	0/69 (0)	0/69 (0)	87.0
Surgery	Final result breast surgery	0/739 (0)	1/739 (0.1)	0/739 (0)	95.9
	Immediate breast reconstruction	1/739 (0.1)	1/739 (0.1)	0/739 (0)	98.5
	Contralateral surgery for symmetry	3/739 (0.4)	1/739 (0.1)	0/739 (0)	98.6
	Contralateral prophylactic surgery	6/739 (0.9)	1/739 (0.1)	0/739 (0)	96.2
	Sentinel node biopsy (SNB)	0/739 (0)	2/739 (0.3)	1/739 (0.1)	95.4
	Axillary surgery	0/739 (0)	1/739 (0.1)	1/739 (0.1)	90.9
	Final result axillary surgery	0/701 (0)	7/739 (0.9)	2/739 (0.3)	89.8
	Reoperative breast surgery	1/739 (0.1)	1/739 (0.1)	0/739 (0)	98.51
	Residual disease detected after reoperation	2/63 (3.2)	5/63 (7.9)	1/63 (1.6)	82.5
	Reoperative axillary surgery	1/739 (0.1)	5/739 (0.7)	0/739 (0)	96.9
	Reason for reoperative axillary surgery	0/33 (0)	9/33 (27)	0/33 (0)	57.6
	Reoperative surgery due to complications	2/739 (0.3)	2/739 (0.3)	0/739 (0)	96.6
Histopathology	Basis for histopathological diagnosis	3/799 (0.4)	8/799 (1.0)	1/799 (0.1)	97.7
	Invasiveness	6/799 (0.8)	21/799 (2.6)	4/799 (0.5)	86.0
	Type of invasive carcinoma	2/688 (0.3)	10/688 (1.5)	1/688 (0.1)	89.7
	Type of in situ carcinoma	2/465 (0.4)	23/465 (4.9)	2/465 (0.4)	87.5
	Number of invasive tumors	7/644 (1.1)	5/644 (0.8)	0/644 (0)	93.6
	Multifocality	9/111 (8.1)	5/111 (4.5)	2/111 (1.8)	76.6
	Number of sentinel nodes	1/604 (0.2)	2/604 (0.3)	0/604 (0)	91.0
	Metastasis in SN	0/604 (0)	12/604 (2.0)	0/604 (0)	93.9
	Number of SN with macrometastases	2/119 (1.7)	23/119 (19)	1/119 (0.8)	85.3
	Number of SN with micrometastases	4/119 (3.4)	41/119 (34)	2/119 (1.7)	82.9
	Number of SN with submicrometastases	2/119 (1.7)	53/119 (44.5)	1/119 (0.8)	89.2
	Nottingham Histological Grade or Nuclear Grade	40/786 (5.1)	50/786 (6.4)	24/786 (3.1)	88.9
	ER status	41/786 (5.2)	40/786 (5.1)	10/786 (1.3)	90.5
	PR status	45/786 (5.7)	48/786 (6.1)	10/786 (1.3)	85.2
	KI67 status	85/786 (10.8)	73/786 (9.3)	85/786 (1.8)	73.5
	HER2 status	60/799 (7.5)	84/799 (10.5)	32/799 (4.0)	73.3
	Presence of vascular invasion	161/786 (20.5)	175/786 (22.3)	99/786 (12.6)	79.8
Postoperative treatment	Postoperative multidisciplinary conference	3/739 (0.4)	9/739 (1.2)	1/739 (0.1)	94.2
	Planned postoperative adjuvant treatment	1/739 (0.1)	9/739 (1.2)	1/739 (0.1)	96.6
	Postoperative radiotherapy	1/673 (0.1)	12/673 (1.8)	1/673 (0.1)	95.5
	Radiotherapy to breast or chest wall	0/519 (0)	0/519 (0)	0/519 (0)	76.9
	Locoregional radiotherapy	0/519 (0)	0/519 (0)	0/519 (0)	87.1
	Endocrine treatment	2/673 (0.3)	14/673 (2.1)	1/673 (0.1)	93.8
	Antiestrogen	0/532 (0)	0/532 (0)	0/532 (0)	81.6
	Aromatase inhibitor	0/532 (0)	0/532 (0)	0/532 (0)	86.8
	Postoperative chemotherapy	2/673 (0.3)	12/673 (1.8)	1/673 (0.1)	94.8
	Anthracycline based	0/232 (0)	0/232 (0)	0/232 (0)	62.5
	CMF	0/232 (0)	0/232 (0)	0/232 (0)	94.8
	Taxanes	0/232 (0)	0/232 (0)	0/232 (0)	77.6
	Other chemotherapy	0/232 (0)	0/232 (0)	0/232 (0)	87.9
	Targeted therapy	4/673 (0.6)	14/673 (0.2)	1/673 (0.1)	95.5
	Trastuzumab	0/59 (0)	0/59 (0)	0/59 (0)	71.2
	Castration (medical)	6/670 (0.9)	20/673 (3.0)	9/673 (0.3)	96.3

A detailed summary of each variable included in the validation can be found in the publicly available report (Swedish only) [12].

### Lead times

**Fig. 2:**
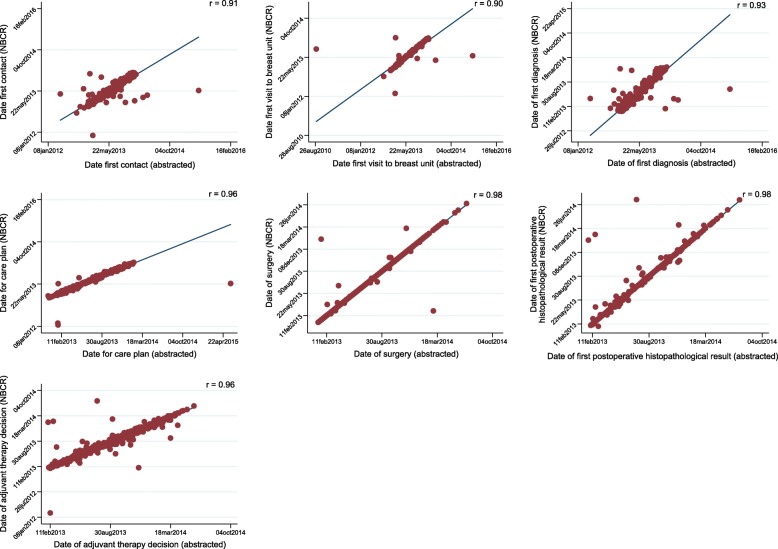
Correlation of information related to date variables recorded in the National Breast cancer register (NBCR) in Sweden and validated data obtained through medical records in 799 women diagnosed with breast cancer between September 2013 and January 2014

The recorded variables (“Date first contact”, “Date first visit to breast unit”, “Date of first diagnosis”, “Date for care plan”, “Date of surgery”) were close to complete in the NBCR (≥ 99.9%). There have been historical ambiguities regarding the definition of the variable “Date first contact” which refers to the date of first contact with the specialist clinic/breast unit, but the extracted data in this material correlated highly with the information recorded in the NBCR (r = 0.91) (Fig. 2). Overall, the correlation coefficients were 0.90 or higher for all lead time variables referring to dates prior to start of treatment.

The completeness of the variables “Date of first postoperative histopathological result” and “Date of adjuvant therapy decision” in the NBCR was slightly lower (92.5 and 92.7%, respectively). However, for both variables the correlation with the information recorded in the medical records was high (r > 0.95).

### Diagnostics

**Fig. 3:**
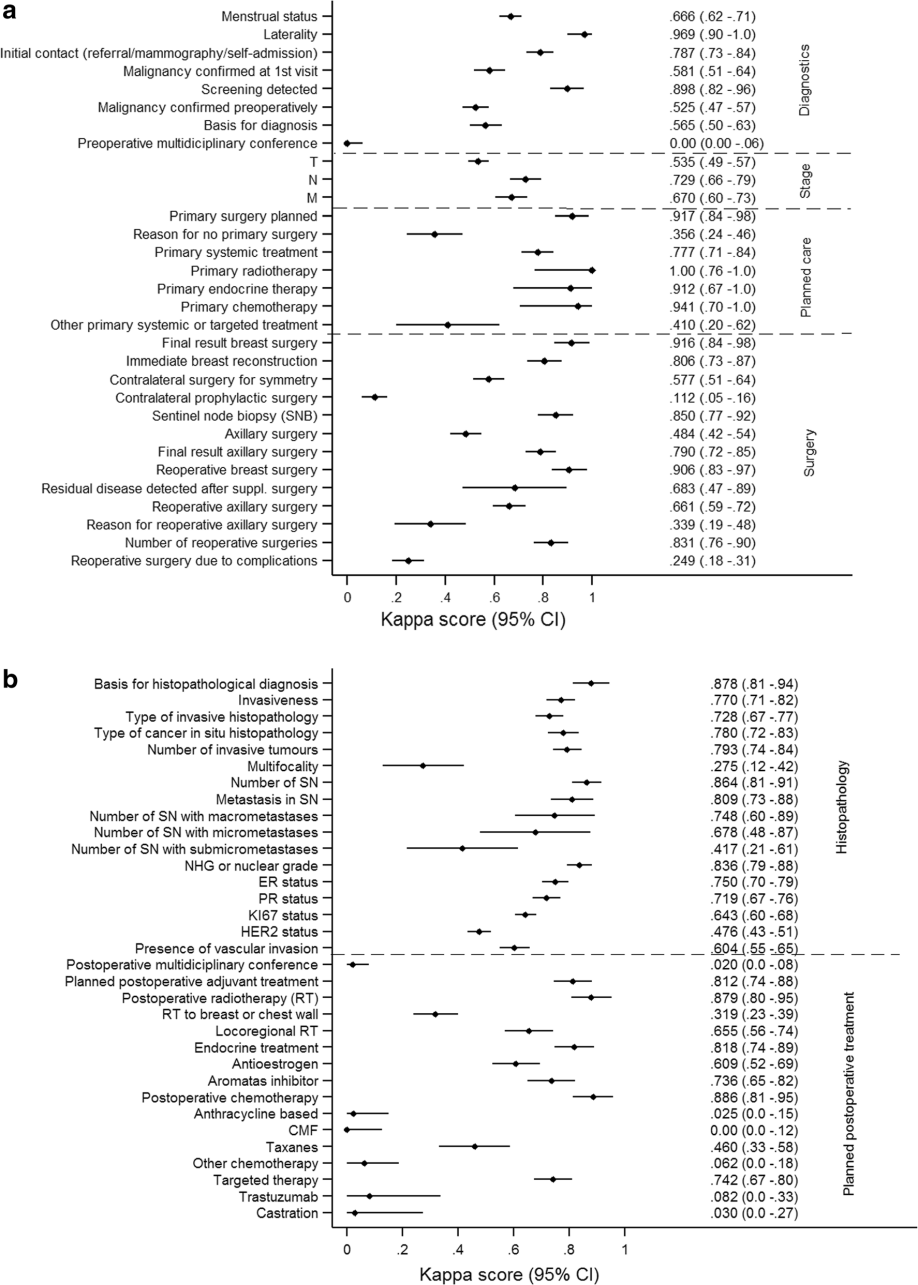
**a** Concordance, as measured by the Kappa score and 95% confidence intervals (CI), in validated variables related to diagnostics, stage, planned care and surgical information. **b** Concordance, as measured by the Kappa score and 95% confidence intervals (CI), in validated variables related to cytopathology and postoperative treatment

While the variable “Detection within the screening program” showed good validity (exact agreement 95%, κ = 0.90, 95% CI: 0.82–0.96), the strength of agreement of variable “Malignant diagnosis verified at first visit (to the breast cancer unit)” was weaker (exact agreement 83%, κ = 0.58, 95% CI: 0.51–0.64) (Fig. 3a).

**Fig. 4:**
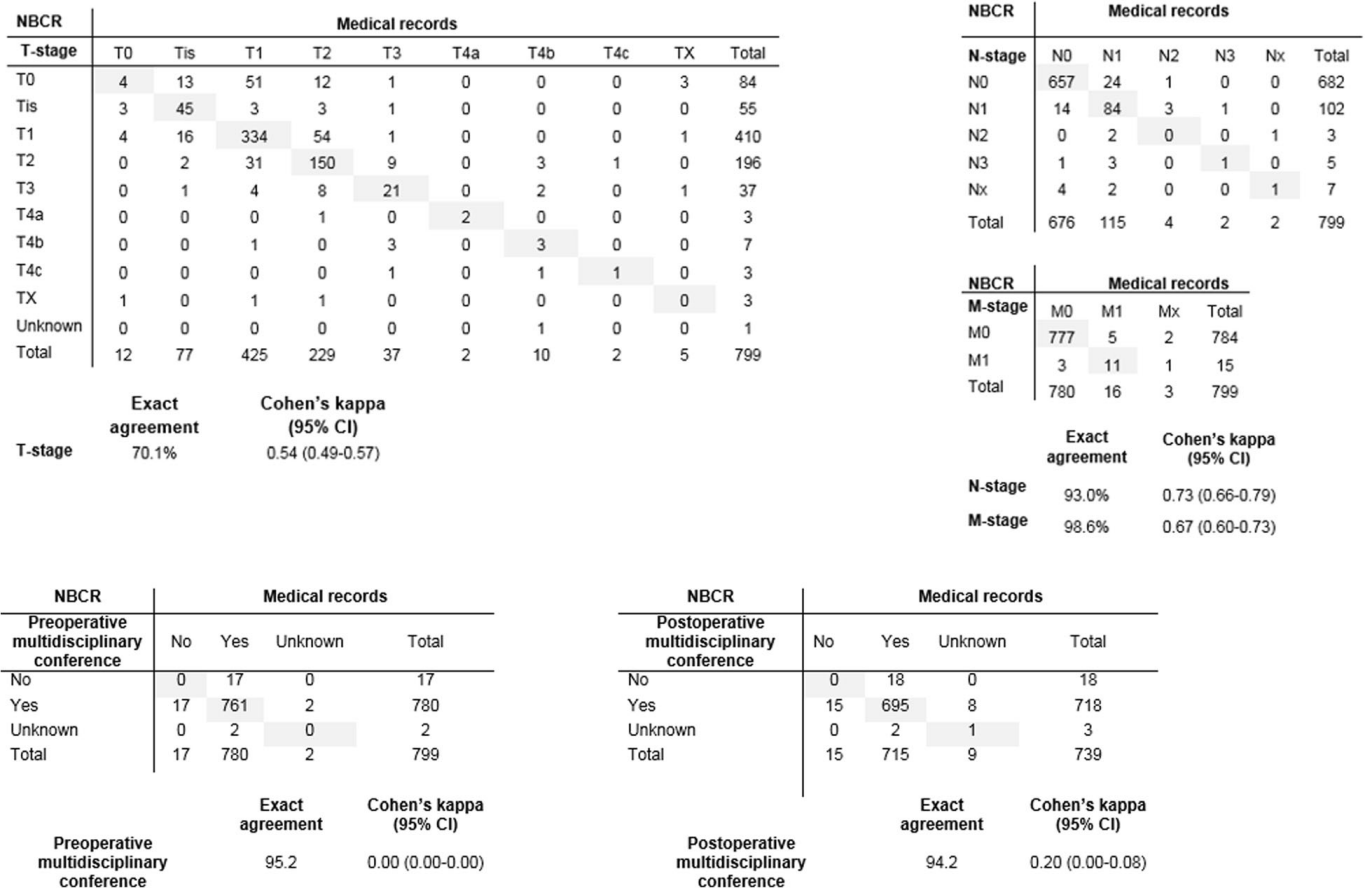
Agreement and concordance between data recorded in the NBCR and the re-abstracted data of variables related to stage (TNM at the time of diagnosis), pre- and postoperative multidisciplinary conference

The exact agreement for the variable “Multidisciplinary Treatment Conference” was high (95%), but the κ zero as 100% of the original records that negated that a multidisciplinary meeting had taken place (*n* = 17) were classified differently in the re-abstracted data (Fig. 4).

### Stage

In order to fulfill the Swedish Cancer Registry requirements the notification form should include information on clinical stage, TNM at the time of diagnosis.

The agreement for clinical TNM classification was moderate although TNM classification is mandatory to report. The T (tumour) category showed lowest agreement (exact agreement 70%, κ = 0.54, 95% CI: 0.49–0.57), followed by the N (lymph nodes) (exact agreement 93%, κ = 0.73, 95% CI: 0.66–0.79) and M (distant metastasis) (exact agreement 99%, κ = 0.67, 95% CI: 0.60–0.73) categories (Figs. 3a and 4).

### Planned care

Recommended primary treatment (surgery, neoadjuvant chemo-, radio- or endocrine therapy) discussed during the Multidisciplinary Treatment Conference, was reported with good validity. Conversely, indications for non-primary surgical treatment (exact agreement 99%, κ = 0.36, 95% CI: 0.24–0.46) and whether other therapies (exact agreement 99%, κ = 0.41, 95% CI: 0.20–0.62) were planned showed weaker agreement (Fig. 3a).

### Surgery

Final result of breast surgery (breast conservation/mastectomy/subcutaneous mastectomy/no breast surgery) and number of supplementary surgeries showed high validity, with exception for contralateral prophylactic surgery (exact agreement 96.2%, κ = 0.11, 95% CI: 0.10–0.16) (Fig. 3a). For the variable “Axillary surgery” the exact agreement was high (91%) but the strength of agreement was relatively low (κ = 0.48, 95% CI: 0.42–0.54), due to inconsistencies in the re-abstracted data. After re-recording of validation responses (i.e. axillary surgery = “yes” if the re-abstracted number of lymph nodes > 0) the κ increased to 0.89. Reasons for supplementary surgery of the axilla had poor validity (exact agreement 58%, κ = 0.34, 95% CI: 0.19–0.48). The variable coding for complications after surgery (“Additional interventions performed due to surgical complications within 30 days”) exhibited low agreement (exact agreement 97%, κ = 0.25, 95% CI:0.18–0.31).

### Histopathology

While the variables “Invasiveness” and “Type of invasive histopathology” demonstrated high validity, the variable “multifocality” was associated with poor agreement (exact agreement 77%, κ = 0.28, 95% CI: 0.12–0.42) (Fig. 3b). This is likely due to ambiguities in the manual since similar information is coded in the variable “Number of invasive tumors” which shows a good result (exact agreement 94%, κ = 0.79, 95% CI:0.74–0.84).

The validity of receptor status variables, ER (Estrogen receptor) status (exact agreement 91%, κ = 0.75, 95% CI: 0.70–0.79), PR (Progesteron receptor) status (exact agreement 85%, κ = 0.72, 95% CI: 0.67–0.76) and KI67 (antigen KI-67) (exact agreement 74%, κ = 0.64, 95% CI: 0.60–0.68) was good when classified as binary variables (No/Yes). Moreover, the correlation between the percentages of immunohistochemical staining, reported to the NBCR and those recorded in the medical records was also strong (ER:r = 0.97, PR:r = 0.94, KI67:r = 0.97). The agreement for the variable HER2-neu (Human Epidermal Growth Factor receptor 2) was, however, weaker (exact agreement 73%, κ = 0.48, 95% CI: 0.43–0.51). HER2 analysis is more time consuming and the results arrive later. Failure to report or to make a distinct notification in the patients file about the test result occurred in 116 cases where the validators recorded that a HER2 analysis had not been carried out despite reported as negative. The validity for “HER2-neu” was heterogeneous across all healthcare regions [12]. Regarding the biologic tumor variables, the proportion in situ cases were overrepresented with respect to missingness (proportion of missing in the NBCR is 30–31% for ER, PR, Ki67 and 34% for HER2-neu).

### Postoperative treatment

Planned postoperative treatment variables showed generally poorer validity (Fig. 3b).

## Discussion

Register data contains an abundance of valuable information useful for improvement of care on a population basis. This validation study showed that data from the Notification form is of high quality and that the validity is generally good. There are to our knowledge few examples of validation concerning process and outcome data of cancer quality registers [13–15]. However, several validation studies are published on national cancer registers [16–19]. Studies based on cancer registries comparing time from diagnosis to treatment between different cancer forms have also been published [20, 21]. An enquiry commissioned by the Ministry of Health and Welfare on, waiting times for diagnosis and treatment derived from three Swedish quality registers found large variations between the different diagnoses breast, colorectal and prostate cancers [22].

Some aspects of the investigated quality dimensions were identified with obvious improvement options. Variables with information considered insignificant were identified. As the registers aim to deliver process data they also serve as a source for clinical research where the demand for detailed information is important. This balance between sufficient and relevant data serving both purposes is challenging. The main findings of each of the four quality dimensions are discussed below.

### Timeliness

For quality purposes and benchmarking improved timeliness is warranted. Lead times measuring waiting times need to be readily available. Timeliness has the potential to alert care givers of short comings in the breast cancer process. On the other hand, many indicators need to be analyzed in significant numbers and over time to make sound conclusions. Lag in completed registrations can be explained in part by the complex adjuvant treatment protocols like primary systemic treatment. Previous reports on cancer register validation from Sweden corroborate our results on deficiency in timely reporting [13, 14]. The observed wide variations in timeliness is assumed to be a consequence of the work flows that differ within the regions and also mirroring diversities in resource allocation. Those units with more efficient workflows could be useful as point of reference. Registration in real time to the NBCR would be the ideal solution and enhance and secure quality of data entries. Efficiency drops when users report retrospectively from medical charts. The optimum would be communicating systems enabling automatic transfer of register data from medical records. An interim solution is allocation of more administrative staff for swift reporting to the NBCR.

### Completeness

The registry’s coverage, when compared against the SCR is high from a national and regional perspective. A slightly lower reporting rate was found in some regions the last year of observation for the period 2010–2014. This reiterates the need for timely reporting, where reported data are displayed online before the monitoring step occurs. The goal is to introduce a structured medical record template for direct transmission of data to the register.

### Comparability

Several items concerning surgery with insignificant information were identified; reason for non-surgical primary treatment and indication for reoperation. Revision surgeries related to postoperative complications was deemed insignificant as only major surgery was a variable. Implementation of standard registration according to i.e. Clavien would be preferable [23, 24]. As morphologic subtype is no longer basis for treatment these variables were also considered redundant. Information regarding planned adjuvant treatment is reported since 2008 as a surrogate for received treatment.

The histopathological report contained data that should be relevant for treatment decision. During the period of 2008–2015 the completeness of pathology reports increased, with more than 80% being adequately reported today due to synoptic reporting. It has both facilitated reporting from the pathologist and reporting to the NBCR due to succinct definitions of variables. Avoiding ambiguous statements in free text reports have increased quality, accuracy, workflow and thereby timeliness in a positive direction. A correct description of the size of the malignancy was challenging and extent of invasive and in situ tumors, numbers of tumors also showed inconsistency between reported and validated information. A revision of the variables giving fewer options would result in further improved quality. The Quality and Standardization Committee in the Swedish Society of Pathology (KVAST) [25] collaborate in the update of guidelines and quality of laboratory analyses. Each individual laboratory should define their own threshold value for receptor status and Ki67 and in the 2015 form “Ki67 status” was recoded according to local cut-off values (Low, High, Not Done, Not available / missing data).

In our experience there are challenges in capturing results of Her2 analysis. To clarify, the results of Her2-neu gene expression when tested with in situ hybridization (ISH) is delayed compared to the rest of the pathology report based on immunohistochemical staining. Our estimation is that when Her2 ISH results arrives later it serves as a basis for therapy recommendation but the care giver has failed in reporting the added information to the register’s report form.

Ambiguities in the manual probably explained the poorer results with respect to consistency found in variables related to adjuvant treatment. In the section related to planned treatment the low consistency was most likely due to failure to distinguish between planned and given treatment in the medical records at reabstraction. Revision of equivocal variables was needed.

### Validity

The proportion of missing values in the database, INCA among the randomly selected patients was lower than 5% for most variables covered by the Notification form. The reported information had generally high exact agreement (> 90%) and/or κ-score. Surprisingly the variable regarding women diagnosed through the screening program, previously considered unreliable, was found to be highly consistent. Assessment of validity has resulted in a thorough review of all variables included in the Notification Form and the project group have proposed clarifications and certain variables to be removed.

### Consequences of the validation study

The following variables were revised: Invasiveness – mixed forms with in situ components were excluded; Histopathological size of cancer in situ – replaced by Extent; Ki67 status - to be reported according to local cut off levels instead of a uniform national cut off level; Recommended postoperative adjuvant treatment – replaced by de facto administered therapy reported in the form Adjuvant treatment which matured with acceptable completeness.

The following variables were omitted: Reason for no primary surgery; Contralateral risk reducing mastectomy; Reason for completion axillary surgery; Reoperation due to early postoperative complication in breast or axilla; Multifocal cancer – as the variable Number of invasive tumors in the breast was more reliable; Lymph vascular invasion.

## Conclusions

Completeness of data, comparability and agreement in the NBCR was high. The current validation has served to revise and omit insignificant variables in the register. Timeliness in reporting showed long lag times which makes data less useful for clinical purposes. The regional differences found are likely explained by variations in workflow. Timeliness seem to be the main challenge. Concomitantly improvements in delivery of real time data have reinforced the impetus to accelerate reporting. In addition, the structured care plan on reducing waiting times in cancer care initiated and implemented in 2016 has also put pressure to improve the process to reach set targets.

## Abbreviations

AKI: Manual from the working group for INCA
EPN: Ethics Committee
ER: Estrogen receptor
HER2-neu: Human Epidermal Growth Factor receptor 2
INCA: Information Network for Cancer care
KI67: Antigen KI-67 is a nuclear protein that is associated with and may be necessary for cellular proliferation
KVAST: Quality and Standardization Committee in the Swedish Society of Pathology
NBCR: The National Breast Cancer Register
PR: Progesteron receptor
SCR: Swedish Cancer Register
SOSFS: National Board of Health and Welfare’s regulations
TNM: T (tumour) N (lymph nodes) M (distant metastasis) Classification of Malignant Tumours
